# A Randomized Controlled Trial Protocol for Using an Accelerometer-Smartphone Application Intervention to Increase Physical Activity and Improve Health among Employees in a Military Workplace

**DOI:** 10.3390/mps5010001

**Published:** 2021-12-21

**Authors:** Emilia Pietiläinen, Heikki Kyröläinen, Tommi Vasankari, Matti Santtila, Tiina Luukkaala, Kai Parkkola

**Affiliations:** 1 Faculty of Medicine and Health Technology, Kauppi Campus, Tampere University, 33520 Tampere, Finland; tommi.vasankari@ukkinstituutti.fi; 2 Special Expert Unit, Centre for Military Medicine, P.O. Box 50, 00301 Helsinki, Finland; 3 Neuromuscular Research Center, Faculty of Sport and Health Sciences, University of Jyväskylä, P.O. Box 35 (VIV), 40014 Jyvaskyla, Finland; heikki.kyrolainen@jyu.fi; 4 Department of Military Pedagogy and Leadership, National Defence University, P.O. Box 7, 00861 Helsinki, Finland; matti.santtila@kolumbus.fi (M.S.); kai.parkkola@tuni.fi (K.P.); 5 UKK Institute for Health Promotion Research, 33500 Tampere, Finland; 6 Research, Development and Innovation Center, Tampere University Hospital, 33520 Tampere, Finland; tiina.luukkaala@tuni.fi; 7 Health Sciences, Faculty of Social Sciences, Tampere University, 33014 Tampere, Finland

**Keywords:** intervention, physical activity, physical performance, accelerometer, mobile applications, sick leave absences, health

## Abstract

Physical activity is beneficial for improving health and reducing sick leave absences. This article describes a protocol for an intervention using an interactive accelerometer smartphone application, telephone counselling, and physical activity recordings to increase the physical activity of workers in the military and improve their health. Under the protocol, employees from six military brigades in Finland will be randomly assigned to intervention and control groups. The intervention group’s participants will use accelerometers to measure their daily physical activities and their quality of sleep for six months. They will receive feedback based on these measurements via a smartphone application. The intervention group’s participants will be encouraged to exercise for two hours per week during working hours, and to participate in telephone counselling. The control group’s participants will continue with their normal exercise routines, without the accelerometer or feedback. The participants of both groups will be measured at the baseline, after the intervention period, and six months after the end of the intervention. The measurements will include accelerometer recordings, biochemical laboratory tests, body composition measurements, physical fitness tests, and questionnaires on sociodemographic factors, physical activities, and health. The primary outcomes will indicate changes in physical activity, physical fitness, and sick leave absences. The findings will help to develop a straightforward and cost-effective model for supporting the health and working capabilities of employees in the military and other workplaces.

## 1. Introduction

Physical activity has many health benefits. It reduces the risk of cardiovascular diseases such as strokes, dyslipidemia, and atherosclerosis. It also reduces insulin resistance and prevents the development of type 2 diabetes. Physical activity also has positive effects on sleep and mental health [1,2]. Obesity increases the risk of type 2 diabetes, coronary disease, high blood pressure, and nonalcoholic fatty liver disease [3]. Visceral fat is a particular health risk [4]. Regular long-term endurance training boosts cellular aerobic energy production and accelerates the oxidation of lipids around the body [5], leading toa reductions in body fat, waist circumference [6], and harmful visceral fat tissue. Physically active members of the working population also have fewer sick leave absences than those individuals who are in poor physical condition [7,8].

Based on surveys and accelerometer measurements carried out among the working population in Finland, the volume of endurance training is not sufficient to sustain the health and physical fitness of the population. Objective data recorded by accelerometers show that 76% of the day is passive time, spent mostly sitting [9]. The poor physical fitness of workers leads to noticeable costs for employers, as well as reduced performance, eliciting the need for physical activation measures.

The trend towards reduced physical activity levels is seen in the military environment, even though soldiers’ good physical performance is essential for success in operations. Therefore, working armies, such as those in the Finnish Defence Forces (the FDF), set special demands for physical fitness. According to law, professional soldiers in the FDF must maintain good physical fitness to meet the demands of their work. For civilian workers in the FDF, maintaining good physical fitness is optional, but the employer can require good physical performance in demanding tasks and peacekeeping operations [10]. A wide survey done [11] among employees of the FDF revealed insufficient exercise habits, with only 51% of the employees in the FDF exercising three times a week.

Reduced physical activity levels increase the risk of metabolic diseases, cardiovascular diseases, and other diseases [1], resulting in increased human resources costs. In the military workplace, Kyröläinen et al. [12] reported that poor muscle fitness and endurance, as well as a high body mass index (BMI), are risk factors for productivity loss, causing additional costs for employers. In 2016, the FDF estimated that sick leave absences cost 35 million euros [13].

To maintain the required physical performance of its workers, the FDF has developed a set of guidelines. To activate workers, one measure in the guidelines is to offer the opportunity to exercise during work for two hours per week, if annual fitness tests are performed [10]. Positive results have previously been achieved using physical training during the workday, showing that these FDF guideline measurements are a step in the right direction [8,14,15,16].

A limited number of studies have been published concerning physical activity and its effects on health in military populations. Additional actions are needed to promote the health and exercise habits of military workers. The present study aims to develop an intervention protocol to increase the physical activity of workers in military settings. Promising results have previously been demonstrated in interventions using accelerometers with online feedback and smartphone techniques in adolescents and adult civilians [17,18,19,20]. In the present study, the use of an interactive accelerometer with a smartphone application, telephone counselling, and the possibility of exercising for two hours a week during working hours to increase participants’ daily activities has been developed. The objectives of this study are to improve health outcomes, reduce stress, improve the quality of sleep, and improve the work performance of employees. The study also investigates whether increased physical activity during the intervention will improve physical fitness and lead to reduced sick leave absences. Thus, the hypothesis of the present study is that the intervention will result in increased daily physical activity, improved physical fitness, health promotion, and reduce sick leave absences. Comparing the changes in the measured parameters between the intervention and control groups will enable the effects of the intervention to be assessed.

## 2. Methods/Design

### 2.1. Participants

The participants will be recruited from six brigades in Finland. Information about the study will first be delivered in an online presentation via the intranet. Later, real-life presentations and written information will be provided to participants. Each civilian and military worker in the FDF, aged between 19–60 years, can be accepted for participation in the study, subject to health considerations including fitness tests and regular occupational health care examinations. Exclusions based on health will be determined following an occupational physician’s examination. Voluntary participants who meet the inclusion criteria will be asked to give their written consent before taking part in the baseline measurements.

### 2.2. Randomization

**Figure 1:**
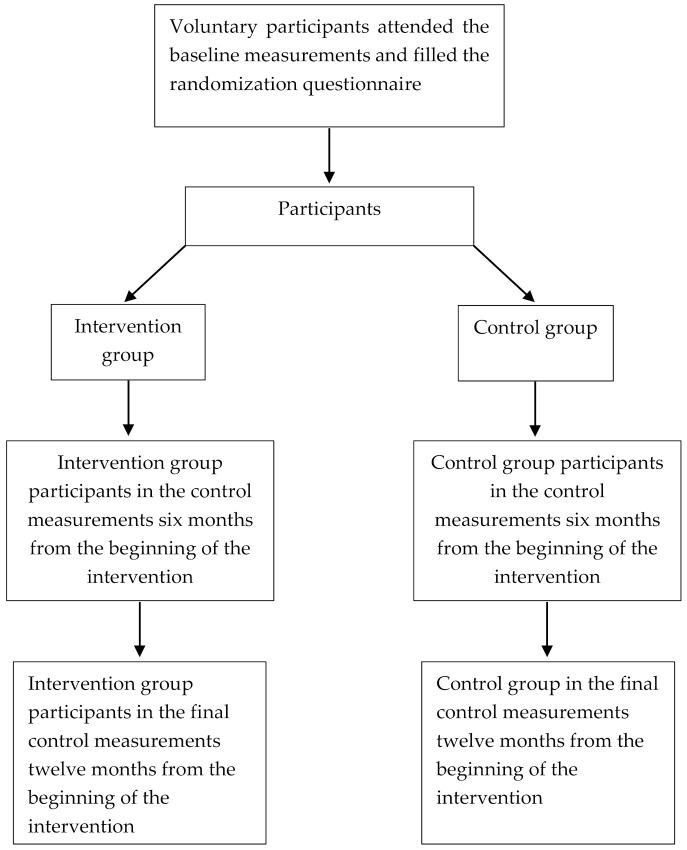
Participants in the different phases of the study.

The study’s participants will be asked to complete a randomization questionnaire. To maximize participation, two-thirds of will be assigned to an intervention group and one-third to a control group (Figure 1). The questionnaire will ask participants to provide personal information to establish homogenous groups according to age, sex, working status (civilian worker vs. soldier), and physical activity. The type of smartphone or other smart device in use will be determined via the questionnaire. Our assumption is that the type of smartphone will not create bias in the results, and this assumption will be confirmed when the results are analyzed. Participants in the intervention group will be asked to confirm that they have a device that supports the application used in the research setting.

### 2.3. Power Calculation and Sample Size

Before the intervention begins, power calculations will be determined, based on the means and standard deviations of personal fitness indices and short sick leave absences (i.e., seven or fewer calendar days) of FDF personnel. According to the results of an endurance test and a muscle strength test, participants’ endurance and muscle strength indices will be determined, using age-dependent reference values. The fitness index will be calculated as an average of the endurance and muscle strength indices [10]. The calculations will be based on the assumption that sick leave absences would reduce as fitness indices increase by a certain amount, i.e., that sick leave absences will reduce by 0.35 days per year for professional soldiers and by 0.40 days per year for civilian workers. The fitness indices will be assumed to increase by 0.14 for male soldiers; 0.45 for female soldiers; 0.34 for male civilian workers; and 0.24 for female civilian workers. For the evaluation of physical fitness, an appropriate number of study participants is approximately 175 soldiers and 99 civilians. To evaluate sick leave absences, an appropriate number of participants is approximately 115 soldiers and 97 civilians (G*Power 3.1.9.2, Wilcoxon signed-rank test (matched pairs), correlation between groups assumption r = 0.80).

### 2.4. Intervention

The intervention will use a technique involving an interactive accelerometer, smartphone application, telephone counselling, and daily physical activity recordings. The intervention will last six months, and measurements of the intervention participants will be performed at the baseline, immediately after the intervention, and six months after the intervention. The intervention procedure is described in Table 1.

#### 2.4.1. Intervention Group

##### Accelerometer Measurements during the Intervention and Smartphone Application Feedback

For six months, the intervention group will wear an interactive accelerometer (Movesense, Suunto, Finland) and receive information on their daily physical activities and quality of sleep via a smartphone application (ExSed, UKK, Terveyspalvelut Oy, Tampere, Finland) The accelerometer will be downloaded with ExSed algorithms, with a mean amplitude deviation MAD and angle for posture estimation APE that have been developed in the UKK Institute (Tampere, Finland). The MAD-APE algorithms can differentiate reliably between sitting and standing [21,22,23]. The accelerometers will be worn on participants’ hips during waking hours and on their wrists during sleep. The accelerometer will transmit the collected physical activity information and sleep data to the ExSed application via Bluetooth. The ExSed application will provide constant feedback on users’ physical activities (e.g., light and moderate activity, steps), sedentary time (e.g., sitting, standing), and quality of sleep (total sleep time, restless sleep time, restful sleep time). The recorded data will be saved to a cloud service [24].

##### Telephone Counselling

The intervention group participants will receive telephone counselling on one occasion during the six-month intervention, providing feedback about their exercise habits. During the counselling session, which will last 10 min, participants will be interviewed about the progression in their exercise habits and hindering factors. The counsellor will guide the participants in finding solutions to existing problems. The telephone counselling sessions will be carried out by two physical education instructors and a research physician, using a pre-planned interview sheet.

##### Training Diary

The intervention group’s participants will be encouraged to exercise two hours per week during working hours, a benefit that is offered to employees under existing FDF guidelines. A training diary will be used to assess these workouts and to differentiate them from the collected accelerometer data. Participants will note in their diaries when they have exercised during working hours (Excel worksheet). The duration and mode of the exercises will be reported.

#### 2.4.2. Control Group

The control group’s participants will continue their normal exercise routines after the baseline measurements, without an accelerometer or feedback. They can benefit from exercising during working hours, but they will not be specifically encouraged to do so, and no exercise diaries will be required.

### 2.5. Measurements

All participants will be subject to baseline measurements at the beginning of the study, and similar measurements at the end of the study. Following the intervention, the intervention group participants will again be subject to all these measurements to observe the effects of the intervention. The control group participants will be subject to physical activity measurements via an RM42 accelerometer for two weeks, to examine whether any changes in their physical activities have taken place due to participation in the study, even without taking part in the intervention.

#### 2.5.1. The Questionnaire

The study’s participants will be asked to answer a questionnaire with topics concerning their lifestyle habits, stress, physical health, and mental health. The questions have been previously validated in various studies (see below). Participants will be asked to fill in the questionnaire online, accessing it from a link sent to them by e-mail. The lifestyle questions will inquire about usage of tobacco products (e.g., cigarettes and snus), alcohol consumption, eating, sleeping, and physical activity. To survey their dietary habits, participants will be also asked about their consumption of different food products during the previous week [25]. The sleep questions will be concerned with the duration of sleeping time, quality of sleep, and interruptions of sleep, as set out in the Nordic Sleep Questionnaire [26]. The questions about physical activity habits will include questions about the participants’ total physical activity, which will be categorized into leisure-time physical activities (LTPA), commuting activities (CPA), and occupational physical activities (OPA) [27]. In addition, inactivity (e.g., screen time), self-reported illnesses, uses of medication, experiences of stress, moods, and motivational factors related to physical activity, education, and working status will be studied [28].

#### 2.5.2. Fitness Tests

The fitness tests will be performed with a protocol similar to the one described in the dissertation of Pihlainen [29]. They will include endurance and muscle fitness testing. All fitness tests, protocols, and techniques will be standardized according to the Fitness Test Manual of the Training Division. The annual fitness tests adhere to the regular protocol in the Finnish Defence Forces: professional soldiers are required to take the tests annually as a prerequisite to field training, and to encourage exercise during working hours; civilian workers are required to take the tests to obtain permission to exercise during their working hours. [10,30]. The tests will be supervised and demonstrated by instructors.

The FDF has three separate tests to evaluate aerobic capacity. Maximal aerobic capacity will be assessed by a 12 min running test [31], a cycle ergometer test [32], or a UKK 2 km walking test [33]. Each participant will select one of those three tests, depending on her/his working status and age. If a participant is unable to perform the 12 min running test, he or she can select either the cycle ergometer test or the UKK 2 km walking test. Each participant will perform only one aerobic test.

Participants will perform the strength and endurance tests on different days to avoid excess strain. Before the maximal test, participants will be asked about the following risk factors: whether there are any personal or close family members (i.e., siblings, parents, or children) who have had coronary disease (e.g., death due to severe cardiac disease, cardiac infarct, cardiac bypass, or assumed or known cardiac disease-related death of a person under 55 years of age); whether the participant has engaged in regular smoking or quit smoking within the previous six months, or had high blood pressure (systolic pressure ≥ 140 mmHg or diastolic pressure ≥ 90 mmHg), high cholesterol (total cholesterol over 5.2 mmol/L, HDL under 0.9 mmol/L or LDL over 3.4 mmol/L), an inactive lifestyle (office work and no regular exercise), high fasting glucose levels (fasting glucose ≥ 6.1), or a BMI of ≥30. If a participant reports two or more risk factors, he or she will be evaluated by a physician. The physician will decide if the maximal fitness test will be carried out. Before the 2 km walking test, the risk evaluation will not be necessary [30].

The 12 min running test will be conducted as a maximal test on an outdoor track during the summer, or in indoor hall during the winter. Participants will be encouraged to run with a maximal effort at a progressively increasing running speed. The test results will be recorded with an accuracy of 10 m [30,31].

A cycle ergometer test will be performed with an indoor cycle ergometer (Ergoline 800S, Ergoselect 100K, Ergoselect 200K, Bitz, Saksa) as a maximal test (for healthy soldiers) or submaximal test (for civilian workers) [34,35]. In the maximal test, a progressive protocol will be used with an initial power output of 50 W, increased by 25 W every 2 min until exhaustion. A participant’s exhaustion will be determined by one or both of the following criteria: (1) a decrease in pedaling frequency to below 60 rpm/min, and/or (2) the participant’s volitional cessation of pedaling. The heart rate of participants will be continuously recorded during the test, using heart rate monitors (Polar Electro, Kempele, Finland). The maximal aerobic capacity (VO_2_max) will be indirectly determined according to the heart rate results. The intraclass correlation has been reported to be high for men with this method (ICC r = 0.82–0.94) [34].

In the UKK 2 km walking test, a 2 km distance will be covered at the maximum speed while the heart rate and performance time are measured. The UKK 2 km test will be used only for civilian workers. The test will be conducted on an outdoor track during the summer and on in an indoor hall during the winter. The time will be recorded to an accuracy of one second, and the participant’s heart rate will be measured at the end of the test. The VO2max will be determined indirectly according to these results [33].

The FDF has three different muscle fitness tests, including standing long-jumps (SLJ), repeated (1 min) sit-ups, and push-ups. The SLJ will be used to assess the power of the participant’s lower extremities. The dynamic muscle endurance capacity of the participant’s trunk and upper extremities will be assessed by sit-ups and push-ups. Together, these three parts of the muscle strength test will demonstrate participants’ overall muscle fitness.

Before the SLJ, participants will be instructed on the proper technique. Three test attempts will be allowed, and the jumps will be performed on a 10 mm thick rubber mattress. The best attempt (i.e., the longest distance from the starting line to the landing point) will be selected for further analysis [36].

Participants will be also shown the proper techniques for both sit-ups and push-ups, and they will be informed that incorrect repetitions will not be accepted for final analysis. The sit-up test will start and end in a lying-down position. In the starting position, participants will lie on their backs as the knees are bent at a 90° angle, with the elbows pointing upwards and the fingers interlocked behind the head. An assistant will support the ankles to keep the heels in contact with the floor during the test. From the starting position, the upper body will be raised forward by the trunk muscles until the elbows reach knee level. One repetition will be completed when the body is lowered and the bottom of the shoulder blades touch the ground. The test result will be expressed as the number of consecutive repetitions in 60 s [37,38].

The push-up repetitions will be counted for a minute, and only consecutive correctly performed repetitions will be accepted for analysis. The correct position for the push-up test will be determined as participants lie on the floor in a front-leaning rest position, with feet parallel at shoulder width and hands positioned so that the thumbs reach the shoulders as the other fingers point forward. The starting position will progress from this position by extending the arms straight while keeping the body in a straight line from the shoulders to the ankles as the knee and hip angles remain steady throughout the test. One repetition will be counted when the torso is lowered by bending the elbows until the upper arms are parallel to the floor and returned to the starting position by extending the arms [38,39].

#### 2.5.3. Body Composition

The body composition measurements of participants will be performed as discussed earlier [38]. The waist circumference measurement, body mass index (BMI), and fat tissue percentage (FAT%) will be used to assess body composition. Body mass (BM) and FAT% will be measured by using the segmental multifrequency bioimpedance analysis assessment (BIA) (InBody 720, Biospace Co Ltd., Seoul, Korea).

The BIA estimates of body composition have been shown to correlate highly with the dual-energy X-ray absorptiometry (DXA) method (r = 0.82–0.95) [40]. The waist circumference of participants will be measured with a tape measure at the level of the iliac crest after exhaling.

Body composition measurement is sensitive to fluid changes in the body. To standardize fluid composition, the measurements will be performed before noon, and participants will be instructed to have fasted for 2 h, avoided alcohol consumption and excess physical strain for 12 h, and empty their bladders before the examination.

#### 2.5.4. Blood Samples and Analyses

Blood samples, for analyzing the health status of participants, will be collected into serum tubes (Venosafe Gel + Clot activator tubes, Terumo Medical Co., Leuven, Belgium) by a qualified laboratory technician during every measurement after a 12 h fast between 07:00 and 09:00. The samples will be centrifuged with 2000× *g* (Heraeus Megafuge 1.0 R, Thermo Scientific, Karlsruhe, Germany) for 10 min at a temperature of +4 °C. The serum will be stored at −80 °C until analysis.

Fasting glucose (GLUC), haemoglobin A1c (HbA1c), and serum insulin will be determined to detect imbalances in glucose metabolism [41,42]. Total cholesterol, high-density lipoprotein (HDL), low-density lipoprotein (LDL), oxidated low-density lipoprotein (ox-LDL), and oxidated high-density lipoprotein (ox-HDL) will be measured to observe body lipid status [43,44,45,46,47]. Alanine transaminase (ALAT), gamma-glutamyl transferase (GT), and carbohydrate-deficient transferrin (CDT) will be measured to evaluate liver function and the possibility of fatty liver disease [48,49,50]. Salivary alpha-amylase will be analyzed as an indicator of elevated stress levels [51].

Plasma glucose (GLUC), serum high-density lipoprotein (HDL), and triglycerides (TG) will be analyzed using a Konelab 20 XTi-device (Thermo Electron Co., Vantaa, Finland), and an isolated LDL fraction will be used for the direct measurement of LDL-cholesterol (CHOD-PAP method). The sensitivity for GLUC is 0.1 mmol/L, and the intra- and inter-assay coefficients of variance are 1.0 and 2.0%, respectively. The ranges for the CHOL, TG, HDL, and LDL assays varies from 0.1–15, 0.09–11, 0.04–2.84, and 0.3–8.9 mmol/L, respectively. Intra- and inter-assay coefficients of variance are 1.1% and 2.1% for CHOL, 1.0% and 3.8% for TG, 3.4% and 3.9% for LDL, and 0.5% and 7.6% for HDL, respectively.

The carbohydrate-deficient transferrin (CDT) will be analyzed using a Capillarys 2 (Sebia Co., Lisses, France) device. The sensitivity for CDT is 0.35%, and the intra- and inter-assay coefficients of variance are 10.3 and 11.2%, respectively.

#### 2.5.5. Baseline and Control Accelerometer Measurements

UKK RM42 accelerometers [9] will be used during baseline and control measurements to analyze daily physical activity and quality of sleep. The accelerometers will be worn for two weeks: on the hip during waking hours and on the wrist during sleep. The aim is that the measurements taken in the second week should demonstrate more physical challenges than the measurements taken in the previous week (i.e., due to field training). Movement in water cannot be measured with the accelerometer. The UKK RM42 accelerometer data will be analyzed subsequently; participants will not receive immediate feedback on the measurements during the actual measurement weeks.

#### 2.5.6. Feedback from the Measurements

All participants will receive feedback from the measurements at different stages of the study. Immediate feedback from the body composition measurements will be provided during every measurement event. Results and feedback from the laboratory tests will be provided after the analyses, and participants will be advised to contact a physician if the test results indicate a need for further examinations or follow-ups. The feedback from the RM42 accelerometer measurements will be provided after the final measurements.

## 3. Results

### 3.1. Primary Outcomes

The primary outcome variables will include changes in accelerometer-measured physical activity, physical fitness test results, and days of sick leave absences. Changes in physical activity levels will be evaluated by comparing physical activity measurements at the baseline and after the intervention. Changes in fitness test results will be evaluated by fitness tests results (i.e., changes in maximal aerobic capacity, SLJ distance, and number of sit-ups and push-ups). Changes in sick leave absences will be evaluated by comparing the number of days of sick leave absences in the year prior to the intervention with the number of days of sick leave absences during the intervention, and with the number of days of sick leave absences one year after the intervention. In addition, each participant’s mean daily number of steps, increases in mean daily total time, increases in number of light physical activities and moderate-to-vigorous physical activities, and reductions in mean daily total time of sedentary behavior will be assessed via two weeks of accelerometer recordings of physical activity at the baseline, immediately after intervention, and six months after intervention.

### 3.2. Secondary Outcomes

The secondary outcomes of the study will be a reduction in the experienced stress assessed by the questionnaire, improvement in the analyzed biomarker levels, reduced BMI, reduced FAT%, decreased waist circumference, and increased amounts of restful sleep when the baseline measurements are compared to measurements taken six months after the intervention.

### 3.3. Reach

Reach will be assessed by dividing the number of the study’s participants by the total number of personnel in the brigades from which the participants will be recruited. Representativeness will be assessed by comparing the baseline information (age, gender, work status) with information obtained from the FDF’s statement of human resources 2018 [52].

### 3.4. Effectiveness

Effectiveness will be assessed by evaluating the shift in the monitored parameters.

### 3.5. Maintenance

The sustainability of the changes will be assessed by measuring the parameters at the baseline, after the six months intervention, and at the end of the study (Table 1).

### 3.6. Statistical Analysis

The continuous distributions will be described using means with standard deviations; regarding categorical data, the number of cases with percentages will be used. Differences between the intervention group and the control group will be tested using an independent samples *t*-test, a Pearson Chi-Square test, or a Fisher’s Exact test. Regression analyses will be used to evaluate the role of physical activity in the changes to physical fitness and the number of sick leave days. Changes in physical fitness and the number of days of sick leave, according to follow-up times and for comparison of the study groups, will be analyzed using general/generalized linear models (univariate, multivariate, and repeated measures) and regression analyses. The drop-out rate can be determined by comparing the number of participants attending baseline measurements to the number of participants attending follow-up measurements. Intention-to-treat analysis will be used to compare changes induced by the implemented intervention of the treatment groups, including all participants as originally allocated after randomization. In addition, a per-protocol analysis will be used to compare changes in the intervention participants who completed the treatment originally allocated. P-values under 0.05 are considered statistically significant. IBM SPSS Statistics version 26.0 for Windows (SPSS Inc., Chicago, IL, USA) was used for statistical analyses.

## 4. Discussion

This article provides an overview of the protocol and evaluation of health and physical training in a study by the Finnish Defence Forces. It is hypothesized that our intervention will increase the physical activity of the study’s participants, leading to reduced sick leave absences, improved fitness test results, reduced stress levels, improved values of the studied health-related biomarkers, reduced waistline circumference and body fat, decreased body mass, and improved reported lifestyle habits. The improved physical activity and health status will increase the readiness of military personnel for response crisis management and other operations, as well as the performance of their daily tasks in the workplace. In addition, an intervention using smartphone application technology to improve physical activity is cost-effective. If promising results are gained among military workers from the intervention, then accelerometer smartphone application feedback technology, combined with feedback from a physical education specialist, could be applied in the general working population to improve exercise habits on a larger scale.

## Figures and Tables

**Table 1:** Intervention procedure and measurements of the study.

	Intervention Group	Control Group
Baseline measurements before the intervention		
Questionnaire	X	X
Body composition measurements	X	X
RM42-Accelerometer measurements	X	X
Fitness tests	X	X
Laboratory tests	X	X
Intervention lasting six months		
Movesense accelerometer measurements and smartphone application feedback	X	
Telephone counselling	X	
Measurements after the intervention		
Questionnaire	X	
Body composition measurements	X	
RM42 accelerometer measurements	X	X
Fitness tests	X	
Laboratory tests	X	
Measurements after six months from the end of the intervention		
Questionnaire	X	X
Body composition measurements	X	X
RM42 accelerometer measurements	X	X
Fitness tests	X	X
Laboratory tests	X	X

## Data Availability

The data presented in this study are available on request from the corresponding author. The data are not publicly available due to the research permit policy of the data owner.
